# „Technology-enhanced learning“ in Anästhesiologie und Notfallmedizin: Ein neuer Weg in der studentischen Lehre im Schatten der Pandemie

**DOI:** 10.1007/s00101-021-01057-9

**Published:** 2021-11-03

**Authors:** Elonka Bergmans, Camilla Metelmann, Bibiana Metelmann, Marie-Luise Rübsam, Felix von Au, Karl-Christian Thies

**Affiliations:** 1 Klinik für Anästhesiologie, Intensiv‑, Notfallmedizin, Transfusionsmedizin und Schmerztherapie, Ev. Klinikum Bethel, Universitätsklinikum OWL der Universität Bielefeld, Campus Bielefeld-Bethel, Burgsteig 13, 33617 Bielefeld, Deutschland; 2 Klinik für Anästhesie, Intensiv-, Notfall- und Schmerzmedizin, Universitätsmedizin Greifswald, Ferdinand Sauerbruchstr., 17475 Greifswald, Deutschland

**Keywords:** Computerunterstütztes Lernen, Anaesthesiologie, Abstandsregeln, Lernen, Medizinstudium, Computer-assisted instructions, Anesthesiology, Physical distancing, Learning, Schools, medical

## Abstract

**Hintergrund:**

Die COVID-19 Pandemie stellt die medizinischen Fakultäten vor beispiellose Herausforderungen. Kontaktbeschränkung als wirksamstes Mittel der Infektionsprävention macht den traditionellen Präsenzunterricht nahezu unmöglich. Daher sind neue Lehrmethoden erforderlich, um das Infektionsrisiko einzudämmen und gleichzeitig hochwertigen Unterricht zu gewährleisten.

**Fragestellung:**

Um den Bedarf an Präsenzunterricht zu reduzieren, wurde unter Anwendung von Open-Source-Software im Rahmen eines Pilotprojekts eine multimediale, virtuelle Lernumgebung für das Fach „Anästhesiologie und Notfallmedizin“ entwickelt und deren Akzeptanz bei Studierenden untersucht. Gleichzeitig beantworten wir die Frage, ob diese Technologie eine taugliche Alternative zum klassischen Präsenzunterricht darstellt.

**Material und Methoden:**

Wir haben mit dem Lernmanagementsystem „Moodle“ eine multimediale eLearning-Plattform entsprechend dem Lehrplan „Anästhesiologie und Notfallmedizin“ für das 2. klinische Studienjahr geschaffen. Es wurde eine anonymisierte Nachkursbefragung mit Multiple-Choice- und Freitextfragen durchgeführt.

**Ergebnisse:**

85,4 % der 157 Teilnehmer bewerteten den Kurs als „sehr gut“, 12,1 % als „gut“ und 1,9 % als „o.k.“. Niedrigere Bewertungen wurden nicht gegeben. 54,8 % bewerteten den Kursinhalt als „sehr relevant“, 43,3 % als „relevant“ und 1,9 % als „neutral“. 94,3 % waren der Ansicht, dass „mehr vergleichbare Online-Kurse angeboten werden sollten“. Die Freitextantworten zeigten, dass Barrierefreiheit und multimediales, selbstgesteuertes Lernen sehr geschätzt wurden. Es wurde jedoch auch angemerkt, dass die praktische Ausbildung nicht durch eLearning ersetzt werden kann.

**Diskussion:**

„Technology enhanced learning“ wurde von unseren Studierenden sehr gut angenommen und als gute Alternative zum Präsenzunterricht bewertet. Für das Erlernen praktischer Fertigkeiten bleibt der Präsenzunterricht jedoch unverzichtbar.

**Zusatzmaterial online:**

Die Online-Version dieses Beitrags (10.1007/s00101-021-01057-9) enthält weitere Tabellen und ein Video zum Schulungsangebot.

## Abstract (en)

**Background:**

Coronavirus disease 2019 (COVID-19) has brought about unprecedented challenges to medical schools. Physical distancing as the most effective means of infection prevention renders traditional classroom teaching nearly impossible and new teaching methods are required to contain the infection risk whilst ensuring high-level education.

**Objective:**

In order to minimize the need for classroom teaching we have created an interactive multimedia eLearning environment using the open-source learning management system “Moodle”. This article describes the development of the eLearning environment and aims to establish the acceptance of technology-enhanced learning (TEL) among medical students and the evaluation of TEL as an alternative to classroom teaching.

**Material and methods:**

We have built a multimedia eLearning environment for fourth year medical students covering the medical school curriculum “anesthesiology and emergency medicine”, which is based on the recommendations of the German Society for Anesthesiology and Intensive Care Medicine (DGAI). We have chosen the open-source learning management system “Moodle” as a platform. “Moodle” is widely used by Anglo-American educational institutions to support and conduct academic and nonacademic teaching. In order to assess the students’ experience, we have carried out an anonymized post-course survey consisting of multiple-choice and free-answer questions.

**Results:**

Of the 157 participants 85.4% rated the course as “very good”, 12.1% as “good” and 1.9% as “OK”. Lower ratings were not given, 54.8% rated the course content as “very relevant”, 43.3% as “relevant” and 1.9% as “neutral”, 94.3% felt that more comparable online courses should be offered. The free-text answers revealed that accessibility and multimedia self-controlled learning were highly valued; however, it was felt that hands-on training cannot be replaced by eLearning.

**Conclusion:**

Technology Enhanced Learning was highly valued by our students and helped to reduce the need for classroom teaching; however, for teaching practical skills classroom teaching remains indispensable.

## Einleitung

Die COVID-19-Pandemie stellt Lehrende an Hochschulen vor neue und unerwartete Herausforderungen. Um die schnelle Ausbreitung der hochinfektiösen Viruserkrankung einzudämmen, mussten Kontakte zwischen Lernenden, Lehrerenden und Patient*innen auf das Notwendigste beschränkt werden. Das resultierende Spannungsfeld ist offensichtlich: Einerseits besteht ein kontinuierlich hoher Lernbedarf, andererseits machen Social und Physical Distancing, als epidemiologische Speerspitzen der Pandemiebekämpfung, Lehre in der herkömmlichen Form nahezu unmöglich.

Im Medizinstudium an der Universitätsmedizin Greifswald ist die Vermittlung von anästhesiologischen und notfallmedizinischen Kenntnissen modular aufgebaut, und theoretische sowie praktische Fertigkeiten werden im Präsenzunterricht gelehrt. Im zweiten klinischen Jahr findet ein einwöchiges Blockpraktikum in Kleingruppen statt. Anästhesiologische Basiskenntnisse werden besprochen, im Simulationszentrum trainiert und anschließend in einer 3‑tägigen OP-Hospitation vertieft. Abgerundet wird dies durch ein interprofessionelles Simulationstraining mit Fokus auf Teamarbeit und Management des kritisch kranken Patienten.

Um die universitäre Lehre unter der Pandemie aufrechtzuerhalten, hat das Zentrum für Patientensicherheit und Simulation der Universitätsmedizin Greifswald als Pilotprojekt ein innovatives „Technology-enhanced-learning“(TEL)-basiertes hybrides Ausbildungskonzept für die studentische Lehre im Fach Anästhesiologie und Notfallmedizin erstellt. TEL ist die Anwendung von Informations- und Kommunikationstechnologie in der Lehre und beim Lernen [11] und umfasst eine breite Palette an Methoden, die von webgestützten Video-Tutorials und interaktiven Apps bis zur „Virtual-reality“-Lernumgebung reichen. Auch die Einbindung von externem, frei im Web verfügbarem Lernmaterial ist möglich.

Mit der Integration von TEL, flankiert von angepasster Simulation [11], haben wir die Theorievermittlung und die interaktive Exploration von Lerninhalten aus dem Seminarraum in die studentische Häuslichkeit verlegt und ein risikoarmes, hybrides Lernumfeld [9] geschaffen. Die hierbei gewonnenen Erkenntnisse werden als Beispiel für die Umsetzung eines solchen Formats in der akademischen Lehre vorgestellt.

In dieser Arbeit beschreiben wir die Kursentwicklung und den Kursaufbau, untersuchen die Akzeptanz von TEL in der studentischen Lehre und beantworten die Frage, ob TEL eine taugliche Alternative zum Präsenzunterricht ist.

## Material und Methode

### Die Lernplattform

Als Lernplattform wurde das Programm Moodle („modular object-oriented dynamic learning environment“, Moodle, West-Perth, Australia, https://moodle.org) verwendet. Moodle ist die führende Lernplattform an europäischen und nordamerikanischen Universitäten [5, 6]. Moodle ist ein webbasiertes, interaktives Open-Source-Programm, das unter der GNU General Public License frei für eine beliebige Anzahl von Teilnehmenden verfügbar ist. Dabei ist jedoch ein Hosting auf einem geeigneten Webserver einschließlich Administration des Betriebssystems und der Datenbanken erforderlich. Dies wurde durch das Rechenzentrum der Universität Greifswald geleistet. Auf der Moodle-Oberfläche können einzelne Kurse und Kurssysteme angelegt und für definierte Nutzergruppen selektiv freigeschaltet werden. Wir haben Moodle gewählt, weil die Lerninhalte interaktiv dargestellt werden können und direkte Kommunikation mit den Studierenden über Diskussionsforen, Blogs und Videokonferenzen möglich ist. Weiterhin können administrative Funktionen wie die Kursverwaltung mit Moodle ausgeführt werden. Die Studierenden können von verschiedenen Endgeräten auf einen Kurs zugreifen (z. B. Desktop, Tablet, Smartphone) [2, 16], was uns besonders wichtig war, da andere Onlinematerialien unseres Zentrums meistens mobil statt über einen Desktop aufgerufen wurden.

### Kursentwicklung und didaktische Überlegungen

Die Lernziele und Kompetenzlevel wurden auf der Grundlage des Nationalen Lernzielkataloges Anästhesiologie der DGAI [3] festgelegt. Eine Übersicht aller Lernziele und Kompetenzniveaus findet sich in Tab. 1.

**Table 1:** 

Thema	Lernziel	Kognitive Lernziele	Stufe/Grad der Komplexität
*Was ist die Anästhesiologie?*			
–	Beschreiben, was das Fach Anästhesiologie beinhaltet	Kenntnisse/Verstehen	1/2
	Die 4 Säulen der Anästhesiologie benennen	Kenntnisse/Verstehen	1/2
*Präoperatives Management*			
–	Die Ziele der präoperativen Visite zusammenfassen	Kenntnisse/Verstehen	1/2
	Die wichtigen Elemente der präoperativen Visite auflisten	Kenntnisse/Verstehen	1/2
*Anästhesiologischer Arbeitsplatz*			
–	Die verschiedenen Komponenten des Basis- und erweiteren Patienten-Monitoring benennen	Kenntnisse/Verstehen	1/2
	Die wichtigen Teile eines Narkosegeräts benennen	Kenntnisse/Verstehen	1/2
*Vorbereitung der Narkose*			
–	Die Wichtigkeit von Checklisten begründen	Analyse	4
	Die verschiedenen Schritte zum Erstellen eines i.v.-Zugangs darstellen	Kenntnisse/Verstehen	1/2
*Einleiten einer Narkose*			
–	Den Ablauf einer Narkoseeinleitung erklären	Anwenden	3
	Die Bedeutung einer Präoxygenierung erkennen	Analyse	4
*Atemwegsmanagement*			
–	Beschreiben, wie Atemwege geöffnet und gesichert werden	Kenntnisse/Verstehen	1/2
	Einige Atemwegshilfsmittel und ihre Anwendung auflisten	Kenntnisse/Verstehen	1/2
*Intraoperatives Management*			
–	Wichtige Medikamente für die Aufrechterhaltung der Narkose benennen	Kenntnisse/Verstehen	1/2
	Erklären, wann verschiedene Formen der Beatmung verwendet werden	Analyse	4
*Komplikationen vermeiden*			
–	Häufige Komplikationen und deren Vermeidung beschreiben	Kenntnisse/Verstehen	1/2
	Einen „anästhesiologischen Klassiker“ kennenlernen	Kenntnisse/Verstehen	1/2
*Narkoseausleitung und Extubation*			
–	Beschreiben, wann und wie eine Narkoseausleitung durchgeführt wird	Kenntnisse/Verstehen	1/2
	Die Extubationskriterien benennen	Kenntnisse/Verstehen	1/2
*Postoperatives Management*			
–	Die Ziele des Aufwachraums definieren	Kenntnisse/Verstehen	1/2
	Erklären, wieso eine strukturierte Übergabe die Patientensicherheit erhöht	Analyse	4
*Erkennen des kritisch kranken Patienten*			
–	Erklären, wie eine Ersteinschätzung des Patienten durchgeführt wird	Erläutern	3
	Das ABCDE-Schema erklären	Synthese	5
*Reanimation*			
–	Beschreiben, wie ein Kreislaufstillstand erkannt wird	Kenntnisse/Verstehen	1/2
	Die Ursachen eines Kreislaufstillstandes benennen	Kenntnisse/Verstehen	1/2
	Die Therapie eines Kreislaufstillstandes erklären	Erläutern	3

Die Lernpfade bilden das breite Spektrum der Anästhesiologie ab und umfassen das peri- und postoperative Patientenmanagement. Ergänzt wird der Kurs durch zwei weitere notfallmedizinische Lernpfade, die die Versorgung des kritisch kranken Patienten sowie lebensrettende Sofortmaßnahmen thematisieren. Eine Übersicht der Lerninhalte und ihre modulare Umsetzung findet sich als Tab. 1 im Zusatzmaterial online (s. Box am Anfang des Artikels).

Die Lernpfade sind ohne festgelegte Reihenfolge über eine zentrale Startseite zugänglich, was eine bedarfsgerechte Bearbeitung ermöglicht und so selbstgesteuertes Lernen unterstützt. Schwerpunktthemen wurden durch Interaktivitäten mit direkten Rückmeldungen hervorgehoben, wodurch Kenntnisfestigung und selbstgesteuertes Lernen gefördert werden.

Für eine optimale Informationsverarbeitung wurden die Lernpfade übersichtlich und gestuft dargestellt. Besonders geachtet wurde auf die Verwendung geläufiger, kurzer Begriffe und kurzer Sätze sowie die Erklärung von Fremdwörtern.

Die Module eines Lernpfads bestehen aus einem Kapitel mit Unterkapitelnavigation und Interaktionen zur Selbstüberprüfung des Gelernten. Das Video (Zusatzmaterial online) gibt eine Übersicht über das Lernprogramm und zeigt den typischen Aufbau eines Kapitels mit einleitendem Text, Lernzielen und Unterkapitelnavigation. In Tab. 2 des Zusatzmaterial online sind alle externen Links aufgelistet. Um die Plattform so abwechslungsreich wie möglich zu gestalten, wird eine Vielfalt unterschiedlicher Medien eingesetzt. So gibt es neben Lernkarten und Multiple-Choice-Fragen auch Interaktionen, bei denen Lückentexte ausgefüllt, Abbildungen zugeordnet oder in die richtige Reihenfolge gebracht oder physiologische Grundlagen in Rechenbeispielen angewandt werden müssen. Für Studierende, die über den dargebotenen Inhalt hinaus eine Wissensvertiefung wünschen, wird diese über weitere Abbildungen, Präsentationen, illustrative Videos und externe Links angeboten.

Die angebotenen externen Links zu Hintergrundinformationen (Tab. 2 des Zusatzmaterial online) (wie wissenschaftlichen Publikationen und aktuellen Leitlinien) wurden im Vorfeld von den Autor*innen im Hinblick auf folgende Qualitätskriterien geprüft: Aktualität, Korrektheit der Inhalte, Neutralität der Darstellung, Produkt- und Barrierefreiheit. Beiträge renommierter Free-Open-Access-Meducation(FOAM)-Seiten [12] werden ebenfalls angeboten. Im Internet frei verfügbare FOAM-Beiträge sind geeignet, sowohl grundlegendes medizinisches Wissen und Praxistipps zu vermitteln als auch im Sinne der „knowledge translation“ wichtige Erkenntnisse von aktuellen Studien, Leitlinien und Kongressen anschaulich zu präsentieren und schnell zu verbreiten [4].

Ein begleitetes Diskussionsforum erlaubt den Studierenden, sich untereinander auszutauschen und direkten Kontakt mit den Tutoren zu suchen, um Fragen zu stellen oder Hintergrundwissen einzuholen.

Dieser asynchrone Kurs wird durch ein synchrones Videoseminar mit jeweils 20 bis 25 Teilnehmenden ergänzt. Hierin konnten die Studierenden im Sinne des „flipped classroom“ [10] mit Unterstützung der Tutoren anhand von Fallbeispielen die erworbenen Kenntnisse explorieren und Lerninhalte gezielt vertiefen.

### Kursevaluation und statistische Auswertung

Um die Akzeptanz der Lernumgebung zu evaluieren, haben wir das Teilnehmer-Feedback ausgewertet. Dies besteht aus einer Kombination von geschlossenen Fragen mit verschiedenen Antwortmöglichkeiten auf einer Drei- bzw. Fünf-Punkte-Likert-Skala und aus offenen Fragen, die sowohl den Inhalt als auch das Format des Kurses betreffen (Zusatzmaterial online: Tab. 3).

Die quantitative Auswertung zu den 4 Themenkomplexen „allgemeine Bewertung“, „Relevanz des Themas“, „Lernzuwachs“ und „technische Probleme“ erfolgte deskriptiv unter Angabe der absoluten und relativen Häufigkeiten. Zur Überprüfung von Zusammenhängen zwischen den genannten 4 Variablen wurde eine loglineare Analyse durchgeführt. Dies erfolgte mittels SPSS® (Statistical Package for the Social Sciences, Version 24, IBM, NY, USA).

Die Codierung und qualitative Auswertung der Freitextantworten erfolgten mit der Software MAXQDA® (Version 20.4.0, VERBI GmbH, Berlin) [13].

Die Teilnehmenden wurden vor dem Ausfüllen darüber informiert, dass die Evaluation freiwillig und anonym ist und die gewonnenen Daten sowohl audit- als auch wissenschaftlichen Zwecken dienen. Auf ein „non-responder tracking“ haben wir verzichtet, da die Evaluation freiwillig war.

Die Ethikkommission der Universitätsmedizin Greifswald hatte keine Bedenken gegen die Studie. Eine berufsrechtliche Beratung war nicht erforderlich.

## Ergebnisse

Insgesamt haben alle Studierende (174) des 8. Semesters die Lernumgebung durchgearbeitet. 157 Studierende (90,2 %) haben an der Evaluation teilgenommen.

### Quantitative Auswertung

**Figure 1:**
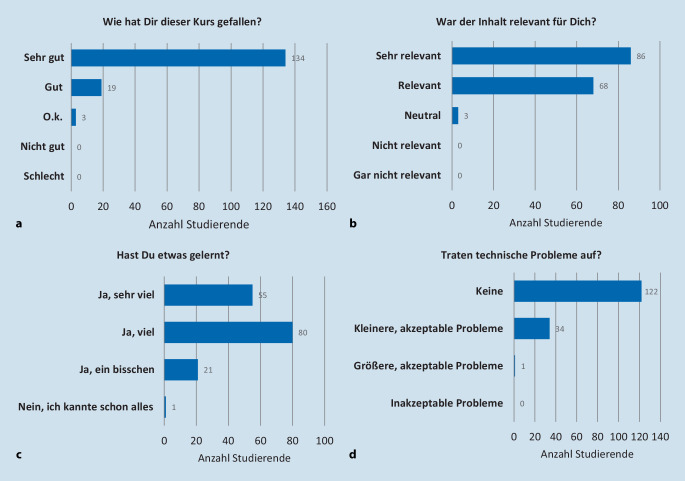

Den Kurs haben 85,4 % (*n* = 134) als „sehr gut“, 12,1 % (*n* = 19) als „gut“ und 1,9 % (*n* = 3) als „o.k.“ bewertet (Abb. 1a). Insgesamt 98,1 % (*n* = 154) schätzten das Kursangebot als „relevant“ bzw. sogar „sehr relevant“ ein (Abb. 1b). „Viel“ bis „sehr viel“ Lernzuwachs gaben 86 % (*n* = 135) der Studierenden an (Abb. 1c). 98,7 % (*n* = 155) der an der Evaluation Teilnehmenden haben den gesamten Kurs durchgearbeitet. Das Onlineformat hat 94,9 % (*n* = 149) „gut“ bzw. „sehr gut“ gefallen; nur 4,5 % (*n* = 7) fanden es „o.k.“. Ein zukünftiges Angebot dieses Onlineformats befürworteten 94,3 % (*n* = 148), während sich 5,7 % (*n* = 9) dagegen aussprachen. Bei 22,3 % (*n* = 35) der Studierenden traten technische Probleme bei der Bearbeitung des Onlinekurses auf, die jedoch durchgehend als „akzeptabel“ bewertet wurden (Abb. 1d).

Die loglineare Analyse der 4 Variablen „allgemeine Bewertung“, „Relevanz des Themas“, „Lernzuwachs“, und „technische Probleme“ ergab, dass es im Modell einen signifikanten Zusammenhang zwischen 2 Variablen gibt, χ^2^(30) = 69,001, *p* < 0,001: der Interaktionen „Lernzuwachs × allgemeine Bewertung“ mit χ^2^(6) = 27,845, *p* < 0,001 und „Lernzuwachs × Relevanz des Themas“ mit χ^2^(6) = 17,010, *p* < 0,009.

Ein signifikanter Zusammenhang zwischen der Variable „technische Probleme“ und den Variablen „Lernzuwachs“ (χ^2^(3) = 5,69; *p* = 0,128) und „allgemeine Bewertung“ (χ^2^(2) = 1,682; *p* = 0,431) wurde nicht gefunden (*p* = 0,431).

### Qualitative Auswertung

#### Inhalt und Verständlichkeit

Der überwiegende Anteil der Studierenden gab an, dass der Inhalt „gut verständlich dargestellt“ wurde. Die Kommentare zeigen, dass die Studierenden die jeweilige Fokussierung der Kapitel erkennen konnten („die Konzentration aufs Wesentliche“) und „die hohe, praktische und praxisrelevante Informationsdichte“ schätzten. Der Einsatz von Hervorhebungen wurde ebenfalls gelobt – „[…] so etwas liebe ich als Student!“

Hinsichtlich der Länge der Texte und Kapitel zeichnete sich ein gemischtes Bild. Insgesamt 25 Studierende haben dies kommentiert; für 7 waren sie „zu kurz“, während 8 Studierende die Kapitel als „zu lang“ bewerteten. Aus weiteren 8 Kommentaren lässt sich auf eine adäquate Kapitellänge schließen.

Weiterhin positiv evaluiert wurde der unterhaltende Stil der Texte mit persönlicher Ansprache der Studierenden als „angenehmer Schreibstil“ „gepaart mit einer ordentlichen Portion Humor“ und die „witzige, abwechslungsreiche Gestaltung“ „ohne [h]erum zu [r]eden“. Ebenfalls positiv merkten die Studierenden den klinischen Bezug der Inhalte und Fallbeispiele an. Insbesondere das komplexe, den Kurs abschließende notfallmedizinische Fallbeispiel, das mehrere unterschiedliche Interaktionen enthält, wurde als „gutes Durchspielen einer Notfallsituation“ gewürdigt.

Aus den Kommentaren ist ersichtlich, dass Texte und Videos aufmerksam bearbeitet wurden. So wurden 2 divergierende Definitionen eines metabolischen Äquivalents (MET) ebenso wie Rechtschreibfehler und unzureichend eingehaltene Hygienestandards in externen Videos moniert.

### Grafische Elemente und Interaktionen

Die eingesetzten Bilder und Videos wurden regelhaft als „anschaulich“ und „ansprechend“ bezeichnet; so „fördern sie den Lern- und Verständnisprozess“.

Die Interaktionen wurden als aufmerksamkeitsfördernd, Motivations- und Konzentrationshilfe angesehen, da „man selbst gefordert war[,] anstatt sich nur stumpf berieseln zu lassen“. Man schätzte die Möglichkeit zur „Mitarbeit“. Dabei dienten die Quiz, „den Lerninhalt […] zu festigen“, der „direkte[n] Anwendung des erarbeiteten [W]issens“ und auch der Wiederholung.

Mehrfach negativ bewertet wurde eine Interaktion zu den reversiblen Ursachen des Herz-Kreislauf-Stillstandes. Hier mussten die Begriffe (Hypoxie, Hypovolämie etc.) exakt eingegeben werden, um die Interaktion erfolgreich abschließen zu können – „[d]as war nervig“, denn es ziehe eine „frustrierte erneute Eingabe nach sich“ und „ich muss[te] gefühlt den [Te]st 10-mal machen“.

Als negativ wurden von einigen Studierenden „das [B]erechnen der Dosierungen“ und die „Lückentexte“ gewertet.

### Technik

Aus einigen Kommentaren geht hervor, dass browserabhängig manche Inhalte nur eingeschränkt bzw. gar nicht abrufbar waren. Einzelne Interaktionen und externe Links waren von der Art der Endgeräte abhängig: „[d]ie Seiten, zu denen die Links geführt haben, waren nicht auf meinem PC, aber auf einem mobilen Endgerät […] aufrufbar“. Einige Studierende monierten, dass Bilder nur „sehr langsam“ bzw. manche Videos gar „nicht geladen werden konnten“ oder „kurz hingen“.

### Onlinekursangebot als Möglichkeit zum selbstbestimmten Lernen

Die Studierenden betonten überwiegend, dass der angebotene Kurs es ermögliche, den Lernprozess individuell und selbstbestimmt zu steuern. Die Arbeit im „eigenen Tempo“ und die „selbstgewählten Pausen“ führten dazu, „[dass] man ohne Stress und mit gute[r] Konzentration lernen [konnte]“.

### Vergleich mit bekannten Lehrformaten

Die Bewertung des Kurses erfolgte in der Regel über den Vergleich mit bekannten Formaten wie Vorlesungen oder synchronen Onlineseminaren. Dabei stand die Variation, die durch die Kombination verschiedener Elemente erreicht wurde, im Vordergrund („viel besser, als eine Vorlesung es hätte machen können“) und wurde als Abwechslung zu den zahlreihen Webinaren gesehen.

Die Quiz und Interaktionen wurden als „Selbstkontrolle“ oder geeignet zur Festigung der Lerninhalte gewertet und mit positiven Begriffen wie „fair“, „sinnvoll“ oder „mit dem […] Text immer beantwort[bar]“ beschrieben.

### Einschätzungen zum Stellenwert digitaler Medien im Medizinstudium

Einige Studierende übertrugen die Erfahrungen, die sie mit dem Kurs gemacht haben, und schätzen das Potenzial dieser Angebote ein. Dabei wurde mehrfach der Wunsch geäußert, dieses Format „auch [in] Zukunft [zu] nutzen, vielleicht als Vorbereitung auf U[ntersuchungs]-Kurse oder das Blockpraktikum“ oder sogar als „curricular[er]“ Bestandteil der Lehre.

Die Studierenden sahen nicht nur die Möglichkeit, auf diese Weise Lehrveranstaltungen vorzubereiten, sondern dadurch u. U. sogar die Präsenzlehre zu verbessern, indem eine Fokussierung auf die praktischen Anteile erfolgen und damit der resultierende Lernzuwachs gesteigert werden kann, denn „[ü]ber dieses Tool kann man sich in Ruhe mit den [t]heoretischen Inhalten vertraut machen und hat am Ende mehr vom Üben der Maßnahmen bei der Präsenz-Veranstaltung“. Betont wurden ebenfalls die Nutzungsmöglichkeiten zu Ergänzung, Nachbereitung, Wiederholung und zum Nachschlagen von Informationen.

In zahlreichen Kommentaren brachten die Studierenden ihre Wertschätzung für den Onlinekurs zum Ausdruck. Sie lobten mehrheitlich das „Engagement“, die „Mühe“ und die „Kreativität“ der Autor*innen.

Dennoch waren sich die Studierenden einig, dass es sich bei dem Kursangebot zwar um eine geeignete „Lösung für das aktuelle Problem“ handelt, der Kurs aber „keinen Ersatz für praktische Erfahrungen“ darstellen kann.

## Diskussion

Dem Zentrum für Patientensicherheit und Simulation der Universitätsmedizin Greifswald ist es innerhalb weniger Wochen mithilfe von TEL gelungen, die studentische Lehre im Fach Anästhesiologie und Notfallmedizin auf eine hybride Lösung aus einem asynchronen Onlinekursformat, kombiniert mit synchronen Videokonferenzen, umzustellen und auf diese Art und Weise den gesamten theoretischen Unterricht aus dem Seminarraum in den virtuellen Raum zu verlegen.

Trotz voranschreitender Digitalisierung der Lehre in vielen Bereichen gehören die technischen Grundvoraussetzungen, wie eine ausreichende Netzabdeckung oder die Verfügbarkeit geeigneter Endgeräte, ebenso wie personelle Ressourcen und didaktische Kenntnisse bei der Konzeption von digitalen Lehrveranstaltungen zu den kritischen Faktoren für eine erfolgreiche Lehre; nicht nur während der COVID-19-Pandemie.

Die Kursevaluation zeigt, dass die Nutzung von TEL im Kontext eines hybriden Lernangebots von den Studierenden positiv bewertet wird. Die loglineare Analyse bestätigt, dass der subjektiv eingeschätzte Lernzuwachs sowohl mit der Relevanz der Inhalte als auch der allgemeinen Einschätzung des Kurses assoziiert ist.

Die Variable „technische Probleme“ war nicht mit den anderen 3 Variablen assoziiert und hatte somit keinen Einfluss auf den Lernzuwachs und die Resonanz des Kurses. Dies wird durch die qualitative Feedbackanalyse bestätigt. Häufig war den Studierenden die Ursache der technischen Störung bekannt, und sie konnten eigenständig geeignete Lösungswege, wie den Wechsel des Endgerätes, einschlagen. Die Einführung von TEL erfordert somit keine zusätzlichen Investitionen in IT-Infrastruktur und kann in der Regel mit bereits vorhandenem Endgerät durchgeführt werden. Hohe Funktionalität und Systemstabilität sind wichtige Aspekte, denn technische Probleme können die Lernerfahrung erheblich beeinträchtigen und demotivierend auf die Studierenden wirken [15].

Die Kriterien „Lernsteuerung“, „Realitätsnähe“ und „gestufte Hilfen“ wurden von den Studierenden explizit im Rahmen der Evaluation herausgearbeitet und können damit als wesentliche Faktoren für die hohe Akzeptanz des Kursangebotes gewertet werden.

Auffällig war, dass die Studierenden die Struktur- und Prozessqualität des Kurses über Vergleiche mit anderen angebotenen Lehrveranstaltungen einschätzten. TEL im Rahmen von hybriden Lernangeboten stößt auf hohe Zustimmung bei den Studierenden und wird als eine Bereicherung der Lernerfahrung wahrgenommen.

Die Nutzung von TEL ermöglicht sowohl eine „diversitätsgerechte Didaktik“ in heterogenen Lerngruppen als auch ein großes Maß an Flexibilität. Timing und der Ort der Informationsaufnahme werden durch den Lernenden bestimmt [7], wodurch die Verarbeitung des Lerninhaltes den individuellen Bedürfnissen besser angepasst (selbstgesteuertes Lernen) und kognitive Überlastung vermieden werden [14].

Gleichzeitig fordern TEL bzw. entsprechende hybride Lehrangebote ein hohes Maß an Eigenverantwortung, den Lernalltag selbstständig zu gestalten.

Die Lehrenden werden mit der Herausforderung konfrontiert, nicht länger nur „Wissen weiterzugeben“, sondern als „Vermittelnde“ tätig zu sein, die den Lernprozess motivierend und reflektierend unterstützen.

Der damit verbundene personelle, materielle und zeitliche Mehraufwand wurde von den Studierenden realistisch ein- und wertgeschätzt. Eine simple Digitalisierung vorhandener analoger Inhalte zur Verwirklichung digitaler Lehre ist nicht ausreichend. Stattdessen sind Kreativität und Engagement sowie fundierte Kenntnissen in moderner Medizindidaktik zur Gestaltung eines „motivierenden Lehrarrangements“ entscheidend [1, 8].

Die außerordentlich positive Resonanz auf die Lernumgebung signalisiert einen lang erwarteten Umbruch in der Lehre, der vorwiegend durch die Erwartungen und Erfahrungen der Lernenden im Umgang mit moderner Informationstechnologie angetrieben wird [17]. Die durch die COVID-19-Pandemie angestoßenen gesellschaftlichen Veränderungen und die zunehmende Digitalisierung unseres Alltags werden die Entwicklung und Implementierung von TEL-basierten Lernsystemen weiter beschleunigen: Durch ihre hohe Flexibilität und Resilienz ermöglichen sie Lernen jederzeit, überall und unter fast allen Umständen. Damit bietet die Integration von TEL die Möglichkeit, zwei Probleme gleichzeitig zu lösen: Die Modernisierung der akademischen Lehre wird angeschoben, und gleichzeitig werden wir mit dem neuen Format die Lehre während einer Pandemie aufrechterhalten können.

## Fazit für die Praxis

- Technology enhanced learning (TEL) kann im Kontext einer hybriden Lernumgebung den Bedarf an Präsenzunterricht reduzieren und ermöglicht die Fortführung akademischer Lehre unter Pandemiebedingungen.
- Der Transfer von existierenden, curricularen Lerninhalten in eine virtuelle Lernumgebung erfordert fundierte Kenntnisse in moderner Medizindidaktik.
- Die Akzeptanz von TEL unter Studierenden ist hoch.
- Selbstbestimmtes Lernen wird von den Studierenden als besonderer Vorteil gewertet.
- Klinischer Unterricht am Patienten oder im Simulationszentrum bleibt unverzichtbar.

## Supplementary Information





