# Supplementary material for: Technology-enhanced learning in anesthesiology and emergency medicine: A new approach to medical school teaching in the wake of the pandemic
Source: Anaesthesiologie. 2021 Nov 3;71(6):444–51. [Article in German] doi: 10.1007/s00101-021-01057-9 (PMC8564793; doi:10.1007/s00101-021-01057-9)
Supplement: Supplementary file 1 [file 101_2021_1057_MOESM1_ESM.pdf]

Zusatzmaterial zum Beitrag „Technology Enhanced Learning in Anästhesiologie und Notfallmedizin. Ein neuer Weg in der Studentischen Lehre im Schatten der Pandemie“ von Bergmans E, Metelmann C, Metelmann B et al. (2021) in *Der Anaesthesist.*

Beitrag und Zusatzmaterial stehen Ihnen auf www.springermedizin.de zur Verfügung. Bitte geben Sie dort den Beitragstitel in die Suche ein.

Tabelle 1 – Kursübersicht und Lerninhalte

Der Kurs besteht aus interaktiven und nicht interaktiven Elementen. Die interaktiven Elemente wurden in erster Linie zur Wissenskonsolidierung und Selbstüberprüfung eingesetzt.

Inhalt	Element	Umsetzung
**Allgemeines**		
Herzlich willkommen	Textseite	
Ankündigungen	Textseite	
Evaluation des Kurses	Feedback	
Fragenforum als Vorbereitung für Zoom-Konferenz	Forum	
**Was ist Anästhesiologie?**		
Was ist die Anästhesiologie?	Arbeits-material	Texte, Abbildungen, Videos, Links
**Präoperatives Management**		
Präoperative Visite	Arbeits-material	Texte, Abbildungen, Links
Testfragen Präoperatives Management	Interaktion	7 Single Choice Fragen
**Anästhesiologischer Arbeitsplatz**		
Anästhesiologischer Arbeitsplatz	Arbeits-material	Texte, Abbildungen, Videos, Links
Kapnographie-Quiz	Interaktion	8 Lernkarten
EKG-Quiz	Interaktion	9 Lernkarten
**Vorbereitung der Narkose**		
Vorbereitung der Narkose	Arbeits-material	Texte, Abbildungen, Videos, Links
**Narkoseeinleitung**		
Einleiten einer Narkose	Arbeits-material	Texte, Abbildungen, Links
Wieso Präoxygenierung?	Interaktion	Lückentext zum Ausrechnen
Reihenfolge Narkoseeinleitung	Interaktion	5 Bilder in korrekte Reihenfolge bringen
Medikamente zur Anästhesieeinleitung	Interaktion	Lückentext zum Ausrechnen
Atemwegsmanagement	Arbeits-material	Texte, Abbildungen, Videos, Links
Atemwegsmanagement	Interaktion	Fragensammlung (1 drag-and-drop-Frage, 1 Multiple Choice Frage, 1 Auswahlfrage)
**Intraoperatives Management**		

	Intraoperatives Management	Arbeits-material	Texte, Abbildungen, Links
	Komplikationen vermeiden	Arbeits-material	Texte und Abbildungen
	Erkenne die Maligne Hyperthermie	Interaktion	„Find multiple Hotspots“ (die falschen Begriffe finden)
	Narkoseausleitung und Extubation	Arbeits-material	Texte
**Postoperatives Management**			
	Postoperatives Management	Arbeits-material	Texte, Abbildungen, Links
**Erkennen des kritisch kranken Patienten**			
	Ersteinschätzung	Arbeits-material	Texte, Abbildungen, Videos, Links
	ABCDE- Schema	Arbeits-material	Texte, Abbildungen, Videos, Links
	Erkennen des kritisch kranken Patienten	Interaktion	7 Bilder in korrekte Reihenfolge bringen
**Reanimation**			
	Reanimation	Arbeits-material	Texte, Abbildungen, Videos, Links
	Advanced Life Support in der Praxis	Externes Tool	
	EKG-Quiz	Interaktion	9 Lernkarten
	Reversible Ursachen Kreislaufstillstand	Interaktion	Lückentext
	Der präklinische Fall am Bodden	Interaktion	Fallbeispiel mit 9 konsekutiven Fragen (Single Choice und Multiple Choice)

Tabelle 2 - Externe Links

Übersicht über die externen links, die über die Lernplattform aufgerufen werden können.

	**Inhalt**	**Link**
**Was ist die Anästhesiologie?**		
	Aufgaben eines Anästhesisten	https://www.youtube.com/watch?v=JyO-DV6s4OM
	4 Säulen der Anästhesiologie	https://www.anaesthesist-werden.de/
**Präoperative Visite**		
	MET – Metabolisches Äquivalent	https://boa.coach/2019/07/02/met-metabolisches-aequivalent-metabolic-equivalent-of-task/
	Letalität verschiedener ASA-Stadien	https://www.sciencedirect.com/science/article/pii/S174391911500206X?via%3Dihub
	RiskCalculator des American College of Surgeons	https://riskcalculator.facs.org/RiskCalculator/index.jsp
	Evaluation des Atemwegs am Beispiel des Weihnachtsmanns	http://news-papers.eu/?p=6678

**Anästhesiologischer Arbeitsplatz**		
	EKG im Notfall	https://nerdfallmedizin.blog/2018/04/07/ekg-im-notfall-teil1-basics/
	Herzaktion und EKG	https://youtu.be/RYZ4daFwMa8
**Vorbereitung der Narkose**		
	KURZcheck des Anästhesiegerätes	https://www.ai-online.info/images/ai-ausgabe/2019/02-2019/2019_2_75-83_Funktionspruefung%20des%20Narkosegeraetes%20zur%20Gewaehrleistung%20der%20Patientensicherheit%20%20Empf.pdf
	Anlage eines iv-Zugangs	https://youtu.be/prkqwpD-KFA
	Gründe, warum Mandrins zum Verschluss iv-Zugänge obsolet sind	https://www.rki.de/DE/Content/Infekt/Krankenhaushygiene/Kommission/Downloads/Gefaesskath_Inf_Teil2.pdf?__blob=publicationFile
**Narkoseeinleitung**		
	Rapid Sequence Induction	https://boa.coach/2018/01/02/berechtigte-stage-fright-rapid-sequence-induktion-rsi-crush-intubation-ileuseinleitung/
**Atemwegsmanagement**		
	Esmarch-Handgriff	https://youtu.be/3JxuxlEHunc
	C-Griff	http://storm4life.de/wp/lehre/tutorials/maskenbeatmung/
	Durchführung einer Intubation	https://youtu.be/2WHN42WzGJg
	Tipps zur Vorbereitung einer Intubation	https://narkosearzt.wordpress.com/2017/01/21/intubation-intubieren-trick-tipp/
	Studie zur Übungsnotwendigkeit einer Intubation	https://pubmed.ncbi.nlm.nih.gov/22060976/
**Intraoperatives Management**		
	Relevante Medikamente zur Allgemeinanästhesie	https://boa.coach/wp-content/uploads/2019/05/pj-spickerneu.pdf
**Postoperatives Management**		
	Übergabe nach SBAR	https://www.bda.de/files/Februar_2016_-_Strukturierte_Patientenbergabe_in_der_perioperativen_Phase_-_Das_SBAR-Konzept.pdf
**Erkennen des kritisch kranken Patienten**		
	Beispielvideos von Asthmaanfällen	https://youtu.be/m8NRrrCDNFA
	Anwendung des ABCDE-Schemas	https://youtu.be/QdwdNH9crLM
**Reanimation**		
	ERC Leitlinien	https://cprguidelines.eu/
	GRC Leitlinien	https://www.grc-org.de/downloads/GRC-Leitlinien-2015-Kompakt.pdf
	Flowcharts des GRC	https://www.grc-org.de/downloads/ERC-Leitlinien_Poster_2015.zip
	Start-Stop-Prozedur der ALS-Kurse des ERC	https://youtu.be/R7mXqkfXN9k

Tabelle 3 – Evaluationsfragen

Die Evaluationsfragen im Multiple Choice und Freitextformat

**Frage**	***Antwort***				
Wie hat dir dieser Kurs gefallen?	Sehr gut	Gut	OK	Nicht gut	Schlecht
Hast du alle Kapitel durchgearbeitet?	Ja	Nein		Keine Angabe	
Wenn du Kapitel NICHT gelesen hast, welche waren dies?	*Freitext*				
War der Inhalt relevant für dich?	Sehr relevant	Relevant	Neutral	Nicht relevant	Gar nicht relevant
Was hat dir am meisten gefallen?	*Freitext*				
Was hat dir am wenigsten gefallen?	*Freitext*				
Hast du etwas gelernt?	Ja, sehr viel	ja, viel	Ja, ein bisschen	Nein, ich kannte schon alles	Keine Angabe
Wie hat dir das Online-Format (Moodle) gefallen?	Sehr gut	Gut	OK	Nicht gut	Schlecht
Traten technische Probleme auf?	Keine	akzeptable Probleme		inakzeptable Probleme	
Falls technische Schwierigkeiten auftraten: Welche waren dies?	*Freitext*				
Sollen in Zukunft mehr vergleichbare Online-Kurse angeboten werden?	ja		nein		
Weitere Kommentare	*Freitext*				
